# The association between lesion location, sex and poststroke depression: Meta‐analysis

**DOI:** 10.1002/brb3.788

**Published:** 2017-08-30

**Authors:** Ying Zhang, Hui Zhao, Yan Fang, Suishan Wang, Haiyun Zhou

**Affiliations:** ^1^ Department of Neurology The First People's Hospital of Shangqiu Henan China; ^2^ Department of Cardiology The First People's Hospital of Shangqiu Henan China

**Keywords:** meta‐analysis, poststroke depression, sex, systematic review, the stroke lesion location

## Abstract

**Background:**

Poststroke depression (PSD) is a common form of stroke patients. Whether the risk of PSD is influenced by the stroke lesion location and sex remains a matter of debate. The objective of this study was to examine the association between the risk of PSD and the stroke lesion location and sex by performing a systematic meta‐analysis.

**Methods:**

Subgroup analyses were performed according to the time interval after stroke onset to assessment for PSD. A total of 31 reports involving 5,309 subjects (for lesion location analysis) and 5,489 subjects (for sex analysis) suffering from stroke were included in this meta‐analysis.

**Results:**

The pooled odds ratio (OR) of PSD after a left‐hemisphere stroke, compared with a right‐hemisphere stroke was 1.11 (95% confidence interval [CI] 0.82–1.49) and OR of PSD after a male stroke, compared with a female stroke was 0.68 (95% CI 0.58–0.81). Subacute poststroke subgroup (1–6 months) significantly favored PSD occurring after a left hemisphere stroke (OR = 1.50, 95% CI 1.21–1.87). Furthermore, there was a statistically significant association between PSD and female stroke for studies with acute poststroke group (OR = 0.73, 95% CI 0.62–0.86) and subacute poststroke stroke phase (OR = 0.69, 95% CI 0.56–0.86).

**Conclusions:**

This systematic review suggests that patients with left hemisphere stroke may be more susceptible to PSD during subacute phase of stroke and female stroke may be more susceptible to PSD during acute and subacute phase of stroke.

## 1 INTRODUCTION

Poststroke depression (PSD) has been recognized as a common sequela of stroke, with an increased morbidity and mortality (Williams, Ghose, & Swindle, 2004). PSD are approximately 85% of patients with strokes (Robinson & Jorge, 2016), and are associated with more serious functional dysfunction (Cully et al., 2005), delayed rehabilitation outcomes (Gillen, Tennen, McKee, Gernert‐Dott, & Affleck, 2001), as well as social withdrawal after stroke (Feibel & Springer, 1982; Robinson, Starr, Kubos, & Price, 1983). Furthermore, after depression remission over a long period of time, PSD have often a high risk of relapse (Ayerbe, Ayis, Rudd, Heuschmann, & Wolfe, 2011). In the last 40 years, there is a wide dissemination of the idea that the brain lesion location can influence the risk of depression after a stroke.

Although a large number of studies have focused on the association between the presence or absence of PSD and the stroke lesion location, the clinical association remains unclear (Robinson & Jorge, 2016). Robinson and colleagues at Johns Hopkins University first reported the hypothesis that the risk of becoming depressed after stroke onset is associated with the location of brain lesion and originates the concept of PSD (Robinson, Shoemaker, Schlumpf, Valk, & Bloom, 1975), and later left‐hemisphere strokes, especially, lesions of the left frontal region (Mayberg et al., 1988; Parikh et al., 1990; Robinson, Kubos, Starr, Rao, & Price, 1983). Subsequently, a large number of studies attempt to confirm these findings. Some studies have replicated the relationship between PSD and left‐hemisphere brain lesions. In contrast, some have even indicated the opposite effect—that PSD is related to right‐hemisphere brain lesions (MacHale, O'Rourke, Wardlaw, & Dennis, 1998). Recent systematically review studies have also not identified the association between the presence or absence of PSD and the stroke lesion location. Two systematical review and meta‐analyses suggest that there is no relationship between risk of PSD and a specific location of stroke (Carson et al., 2000; Hadidi, Treat‐Jacobson, & Lindquist, 2009). Bhogal and colleagues conduct a systematic review and maintain that PSD is associated with the left brain hemispheric lesion after stroke onset (Bhogal, Teasell, Foley, & Speechley, 2004), while Yu and colleagues report a weak association between PSD and right brain hemispheric lesion after stroke onset (Yu et al., 2004). A recent systematic review suggests the association between PSD and lesion location is affected by stratification of time between stroke and the assessment of PSD, and finds a strong relationship between PSD and right brain hemispheric lesion within subacute poststroke phase (Wei et al., 2015). These variances in the association between PSD and lesion location can be explained by the mixed stroke type (Esparrago Llorca, Castilla‐Guerra, Fernandez Moreno, Ruiz Doblado, & Jimenez Hernandez, 2015; Tsai, Anderson, Thomas, & Sudlow, 2016, 2015) and stratification of gender (Poynter et al., 2009; Mackay & Mensah, 2004). More especially, it is reported that there is a sex‐specific prevalence of PSD and these discrepancies in medical care and rehabilitation are considered to be reflected in a difference in the prevalence of PSD between the sexes (Poynter et al., 2009).Therefore, it is plausible that these variances in the association between PSD and stroke lesion location are reflected in gender effect on the association.

This systematic review extends the related and existed literatures and further explores the potential reasons of the heterogeneity that might exist among results. The principal aim of this study was to investigate the specific association between the presence or absence of PSD and stroke lesion location and sex more precisely and completely.

## 2 METHODS

### 2.1 Searching strategy

This systematic review included all studies in stroke patients with an assessment of depression, which examined the association between the presence or absence of PSD and the stroke lesion location and gender were initially eligible for inclusion.

We first performed a comprehensive searching strategy updated to 19 August 2016, via PubMed, PsycINFO**,** ISI Web of Science, EMBASE, and CINAHL. These keywords in our searching strategy were as follows: “stroke or cerebrovascular or poststroke or poststroke” and “depression or mood disorder or affective disorder or depressive disorder”. The on‐line abstracts were reviewed, and reprints of all potentially eligible studies were obtained.

In addition, we supplemented forward citation searches of key relevant reviews and perused the reference lists of included primary articles and relevant reviews by hand to identify additional citations not identified by the databases.

### 2.2 Study selection

The inclusion criteria used to choose subjects as follows: (1) patients in the studies experienced at least one stroke (over age 15) with men and/or women; (2) the relationship between PSD and stroke lesion location must have been investigated; (3) information sufficient for the computation of effect sizes must have been provided; (4) the studies defining depressive disorder as a diagnosis made using an operationalized definition (Diagnostic and Statistical Manual III, IIIR, or IV, or International Classification of Diseases, ninth or tenth revision); (5) imaging using either computed tomography (CT) or magnetic resonance imaging (MRI) scanning; (6) the studies must have been published in English and involved human subjects.

The exclusion criteria used to choose subjects as follows: (1) studies reported stroke patients in specific locations; (2) studies did reported the results stratified by sex; (3) studies reported stroke patients of a specific age group; (4) studies reported both stroke and head injury unless they reported separate results for stroke patients; (5) We excluded duplicate studies. The duplicate studies were defined by the following criteria: shared sampling frame, similar reported sample characteristics, the same study dates, and the same grant funding numbers. If the study was considered to be duplicate studies we selected the original data on the largest number of subjects. In cases of the same number of subjects, we selected the earliest one; (6) We excluded the only abstracts, case reports, review articles, retrospective recruitment studies or pharmacological intervention studies.

### 2.3 Data extraction

According to the inclusion and exclusion criteria, two reviewers (Zhang Y and Zhao H) extracted data from the selected studies independently. The primary reviewers made the final decision if the discrepancies were found between all authors. The following information in each included study was extracted, including: (1) first author name, (2) published year, (3) demographic characteristics, (4) method of diagnosis of depression, (5) sample size, (6) the time between stroke and assessment of depression, (7) assessment of lesion localization, (8) history of stroke or depression, (9) the source of patients (acute inpatients, rehabilitation units, community), (10) final conclusions. If a study was the lack of the related information required for our meta‐analysis, we made an attempt to contact the corresponding author to obtain the required information not reported.

### 2.4 Quality assessment

Two reviewers (Fang Y and Wang SS) evaluated the quality of included studies according to the Newcastle–Ottawa scale (NOS) independently (http://www.ohri.ca/programs/clinical_epidemiology/oxford.asp). Using a “star” rating system, the NOS assesses the quality of the study according to three aspects, including selection, comparability, and exposure (case–control studies) or outcome (cohort studies).Therefore, The scores of each study were classified into the nine grades, ranging from 0 (worst) to 9 stars (best). The quality of each study was further divided into three grades: low (1–3 stars), intermediate (4–5 stars), or high (6–9 stars). Studies with a score equal to or higher than 4 could only be included into this meta‐analysis.

### 2.5 Statistics analysis

The statistical analysis and forest plots were performed using RevMan 5.1 software (RevMan, 2011). Taking into consideration possible heterogeneity among studies, we first performed a statistical test for heterogeneity using Cochran's Q statistics (*p*_heterogeneity_) and *I* ^2^ metrics (Gu et al., 2013). The pooled odds ratio (ORs) and 95% confidence interval (CIs) were evaluated using a random‐effects model when if the *p*_heterogeneity_ < .10 or *I* ^2^ > 50%, it was considered to be significant heterogeneity; otherwise, we estimated them by a fixed‐effects model. To evaluate the stability of results, we conducted a sensitivity analysis. That is, by, to evaluate whether our results were affected due to removing one study at a time, we analyzed the rest studies and recalculate the pooled OR. We used the Begg's funnel plot and Egger's test to evaluate whether there was a potential publication bias (Begg & Mazumdar, 1994; Egger, Davey Smith, Schneider, & Minder, 1997). The statistical threshold was set at a *p* < .05 expect for Cochran's *Q* test (*p*_heterogeneity_ < .10).

In addition, we performed subgroups analyses to explore potential sources of the heterogeneity based on each study characteristic. First, we included all studies and calculated the relationships of the presence or absence of PSD and stroke lesion location and sex. The results at the first follow‐up period were just included if several results at different follow‐up periods were reported. Second, we conducted subgroups analyses according to the timing of interview for PSD of the included studies. We named ≤1 month as acute poststroke phase, 1–6 months as subacute poststroke phase, and >6 months as chronic poststroke phase (Wei et al., 2015). According to the source of patients (clinic, rehabilitation center, and community), we also conducted a supplementary analysis.

## 3 RESULTS

### 3.1 Characteristics for studies included

We had a initial search and found 4,389 citations. According to the criteria outlined above after screening based on their abstracts, we initially excluded 3,758 citations. After we screened their full texts, we found that 31 original reports included categorical data on both depression and gender and were included in the meta‐analysis (Folstein, Maiberger, & McHugh, 1977; Andersen, Vestergaard, Ingemann‐Nielsen, & Lauritzen, 1995; Herrmann, Bartels, Schumacher, & Wallesch, 1995; Bjerg Bendsen, Bjerg Bendsen, Lauritzen, Vilmar, & Bech, 1997; Kase et al., 1998; Gainotti, Antonucci, Marra, & Paolucci, 2001; Singh et al., 2000; Desmond et al., 2003; Hsieh & Kao, 2005; Nys et al., 2005; Tang et al., 2005; Glodzik‐Sobanska et al., 2006; Caeiro, Ferro, Santos, & Figueira, 2006; Brodaty, Withall, Altendorf, & Sachdev, 2007; Fuentes, Ortiz, Sanjose, Frank, & Diez‐Tejedor, 2009; Nidhinandana et al., 2010; Nishiyama et al., 2010; Tennen et al., 2011; Shi et al., 2015; Choi‐Kwon et al., 2012; Zhang, Pan, Wang, & Zhao, 2013; Rajashekaran, Pai, Thunga, & Unnikrishnan, 2013; Angeleri et al., 1997; Finklestein et al., 1982; Grasso et al., 1994; Ng, Chan, & Straughan, 1995; Sun et al., 2014; Jiang, Lin, & Li, 2014; Saxena & Suman, 2015; Wei et al., 2016; Metoki et al., 2016). The study characteristics and demographics are provided in Table 1.

**Table 1:** Characteristics of eligible studies

Reference	Year	Source of patients	Prevalence of PSD, %	Onset since stroke	Quality assessment by NOS
Folstein et al. (1977)	1977	Rehabilitation	45	30 days	*********
Finklestein et al. (1982)	1982	Rehabilitation	76	Within 2 days	*******
Grasso et al. (1994)	1994	Clinic	53.3	2.5 months	********
Andersen et al. (1995)	1995	Clinic	38.3	Within 7 days	********
Herrmann et al. (1995)	1995	Clinic	22	Within 2 months	******
Ng et al. (1995)	1995	Rehabilitation	55.8	21.6 days	*******
Bjerg Bendsen et al. (1997)	1997	Rehabilitation	15.6	2 weeks	********
Angeleri et al. (1997)	1997	Clinic	29.7	Within 10 days	*******
Kase et al. (1998)	1998	Community	37.9	1 week	*********
Singh et al. (2000)	2000	Clinic	36	3 months	******
Gainotti et al. (2001)	2001	Rehabilitation	76.6	1 month	*******
Desmond et al. (2003)	2003	Clinic	11.2	3 months	*******
Hsieh & Kao (2005)	2005	Clinic	34.3	Not given	*********
Nys et al. (2005)	2005	Clinic	52	Within 3 weeks	*******
Tang et al. (2005) gender	2005	Clinic	16.4	3 months	*******
Glodzik‐Sobanska et al. (2006)	2006	Clinic	31	10 days	********
Caeiro et al. (2006)	2006	Clinic	46	<4 days	*******
Brodaty et al. (2007)	2007	Clinic	27.4	Not given	********
Fuentes et al. (2009)	2009	Clinic	28.8	10 days	********
Nidhinandana et al. (2010)	2010	Clinic	46.5	>6 months	*******
Nishiyama et al. (2010)	2010	Clinic	34.3	1 months	*******
Tennen et al. (*^2011^)	2011	Clinic + rehabilitation	37.3	Within 4 months	*******
Choi‐Kwon et al. (2012)	2012	Clinic	13.7	3 months	*******
Rajashekaran et al. (2013)	2013	Clinic	45.2	<6 months >2 weeks	*******
Zhang et al. (2013)	2013	Clinic	27.5	Within 2 weeks	*******
Sun et al. (2014)	2014	Clinic	31.4	<6 months	********
Jiang et al. (2014)	2014	Clinic	25	2–6 weeks	********
Saxena & Suman (2015)	2015	Clinic	57	Not given	*******
Shi et al. (2015)	2015	Clinic	29	14 days	*******
Metoki et al. (2016)	2016	Rehabilitation	16.9	10 days	*********
Wei et al. (2016)	2016	Clinic	19.3	7 days	*********

Asterisks indicate number of stars awarded for all items in quality assessment by NOS.

NOS, Newcastle–Ottawa scale; POS, post‐stroke depression.

### 3.2 Risk effect of assessment

**Figure 1:**
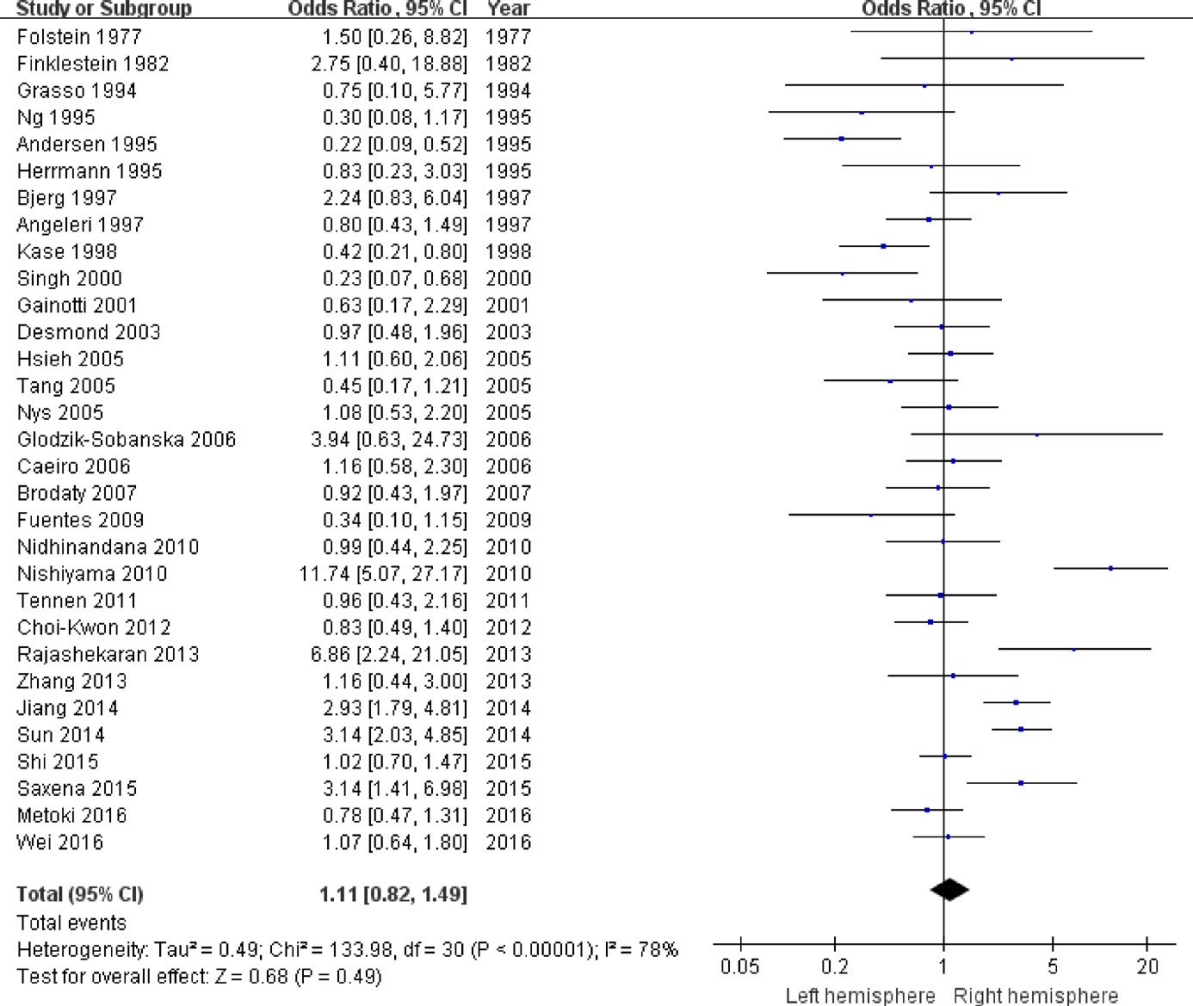
The forest plots of odds ratio with 95% confidence interval for the overall association between stroke lesion location and depression risk

First, all studies were pooled to assess the relationship between the prevalence of PSD and the stroke lesion location. The total 43 studies involved 5,309 stroke patients, including 2,123 left hemisphere stroke and 3,186 right hemisphere stroke. PSD with left hemisphere lesion were 675 patients and PSD with right hemisphere lesion were 963 patients. Because there was a significant heterogeneity (*p*_heterogeneity_ < .00001, *I* ^2^ = 78%, Figure 1), the random‐effects model was used to synthesize the data. The pooled OR with 95% CI was 1.11 (0.82–1.49) for the relationship between the PSD risk and the stroke lesion location (Figure 1).

**Figure 2:**
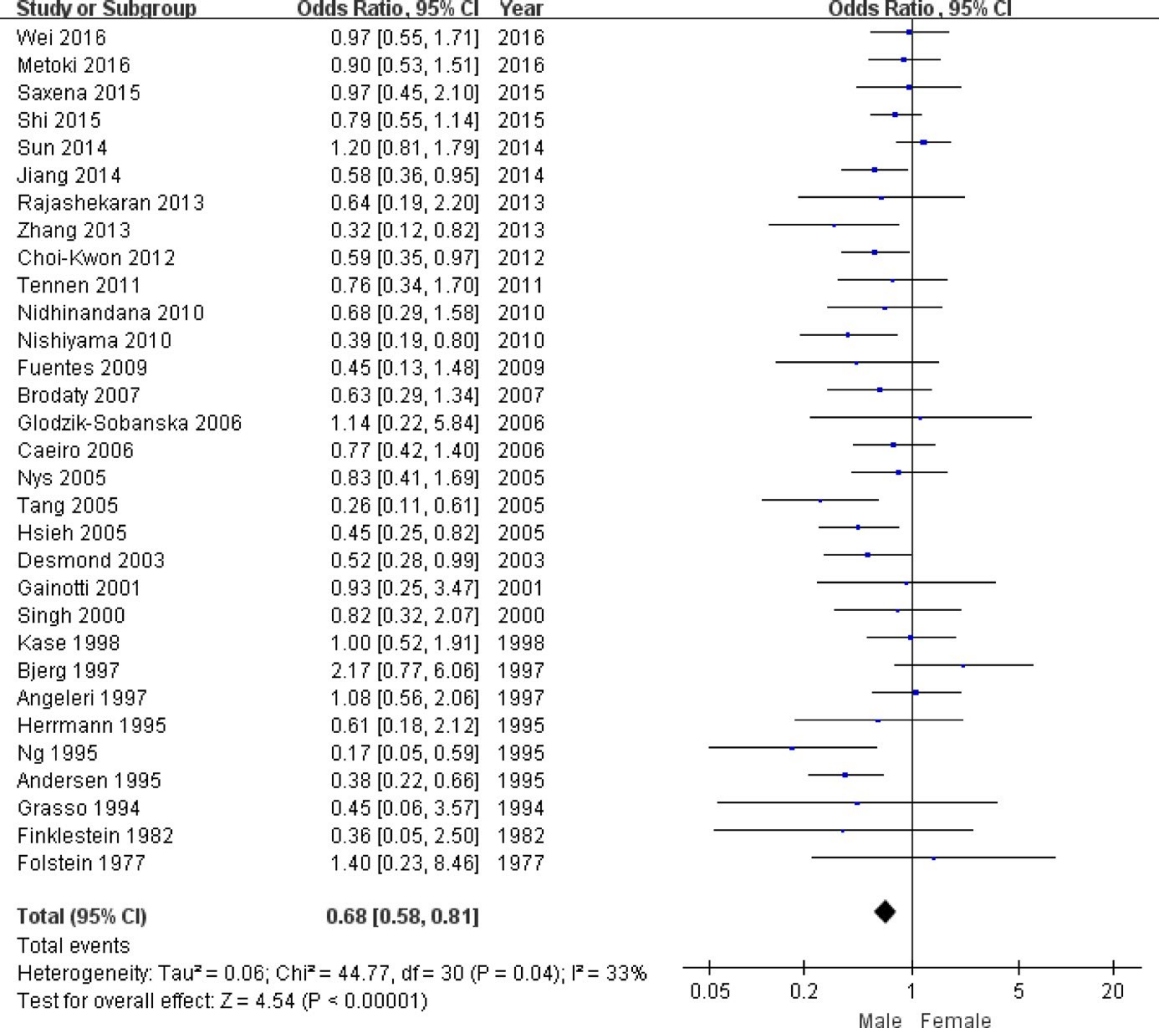
The forest plots of odds ratio with 95% confidence interval for the overall association between gender and depression risk

Second, all studies were pooled to assess the relationship between the prevalence of PSD and the gender. The total 43 studies involved 5,489 stroke subjects, including 3,228 male strokes and 2,261 with female strokes. There were 872 PSD with male patients and 771 PSD with female patients. The random‐effects mode was chose l to synthesize the data due to significant heterogeneity (*p*_heterogeneity_ < .04, *I* ^2^ = 33%, Figure 2). The pooled OR with 95% CI was 0.68 (0.58–0.81) for the relationship between PSD risk and gender (Figure 2).

### 3.3 Source populations

Subgroups analyses were performed according to population source and are provided in SI results and Figures S1–S3

### 3.4 Timing of first interview after stroke

**Figure 3:**
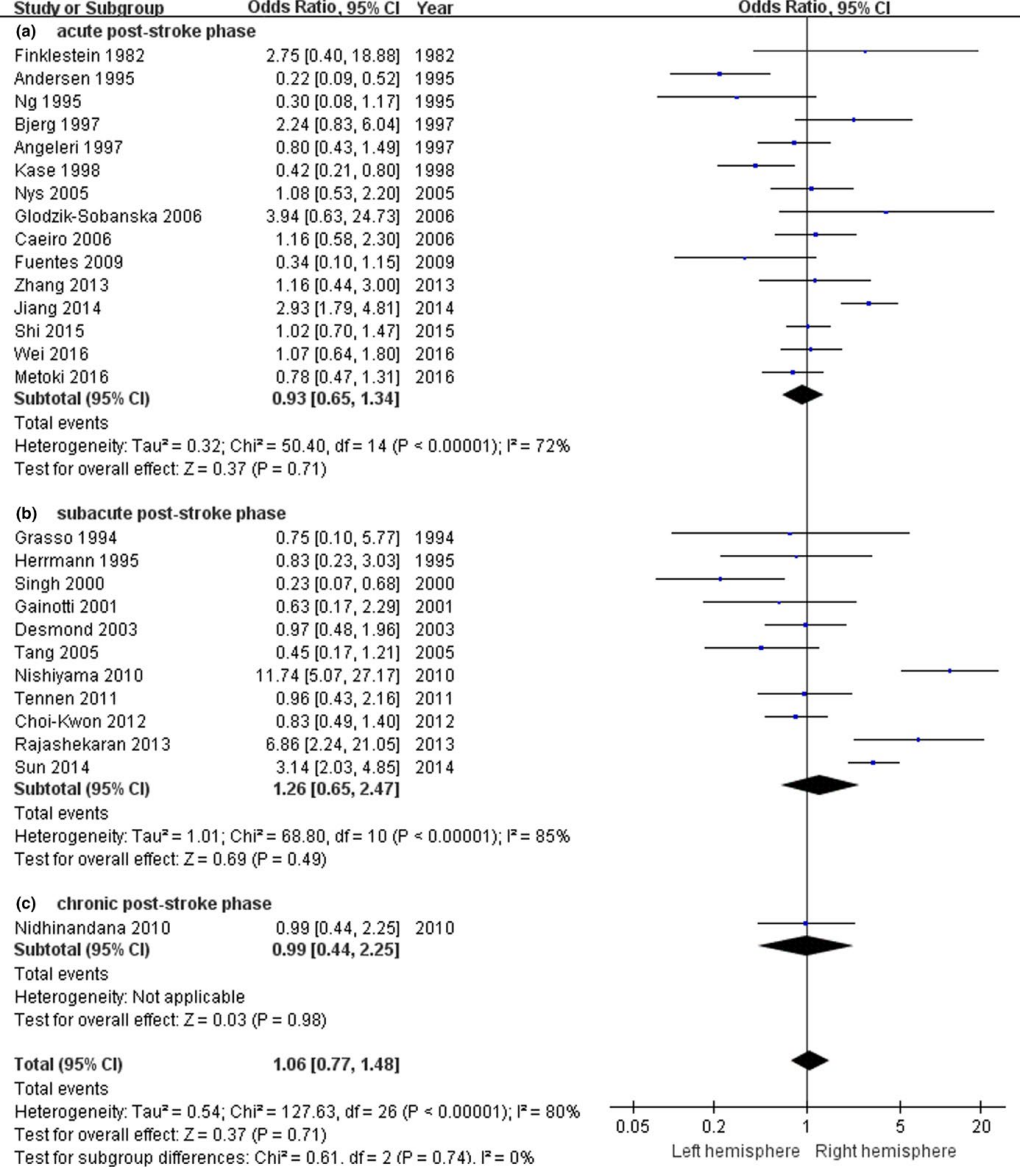
The forest plots of odds ratio with 95% confidence interval for the association between lesion location and depression risk according to time since stroke onset (*n* = 27)

Subgroups analyses were performed according to the timing of interview for PSD. In assessing the relationships between the prevalence of PSD and the stroke lesion location, our results showed that significant heterogeneity was found in acute poststroke group (*n* = 15, *p*_heterogeneity_ < .00001, *I* ^2^ = 72%, Figure 3a), and subacute poststroke group (*n* = 11, *p*_heterogeneity_ < .00001, *I* ^2^ = 85%, Figure 3b). Therefore, the random‐effects model was performed to synthesize the data. There was only a statistically significant association between PSD and left hemisphere stroke for subacute poststroke group (OR = 1.50, 95% CI 1.21–1.87, Figure 3b). Our results indicated that the stroke patients with left hemisphere lesion may be more susceptible to PSD during subacute phase of stroke. Because the study of chronic poststroke group in this meta‐analysis only included 1 study (Figure 3c), it did not reveal any associations.

**Figure 4:**
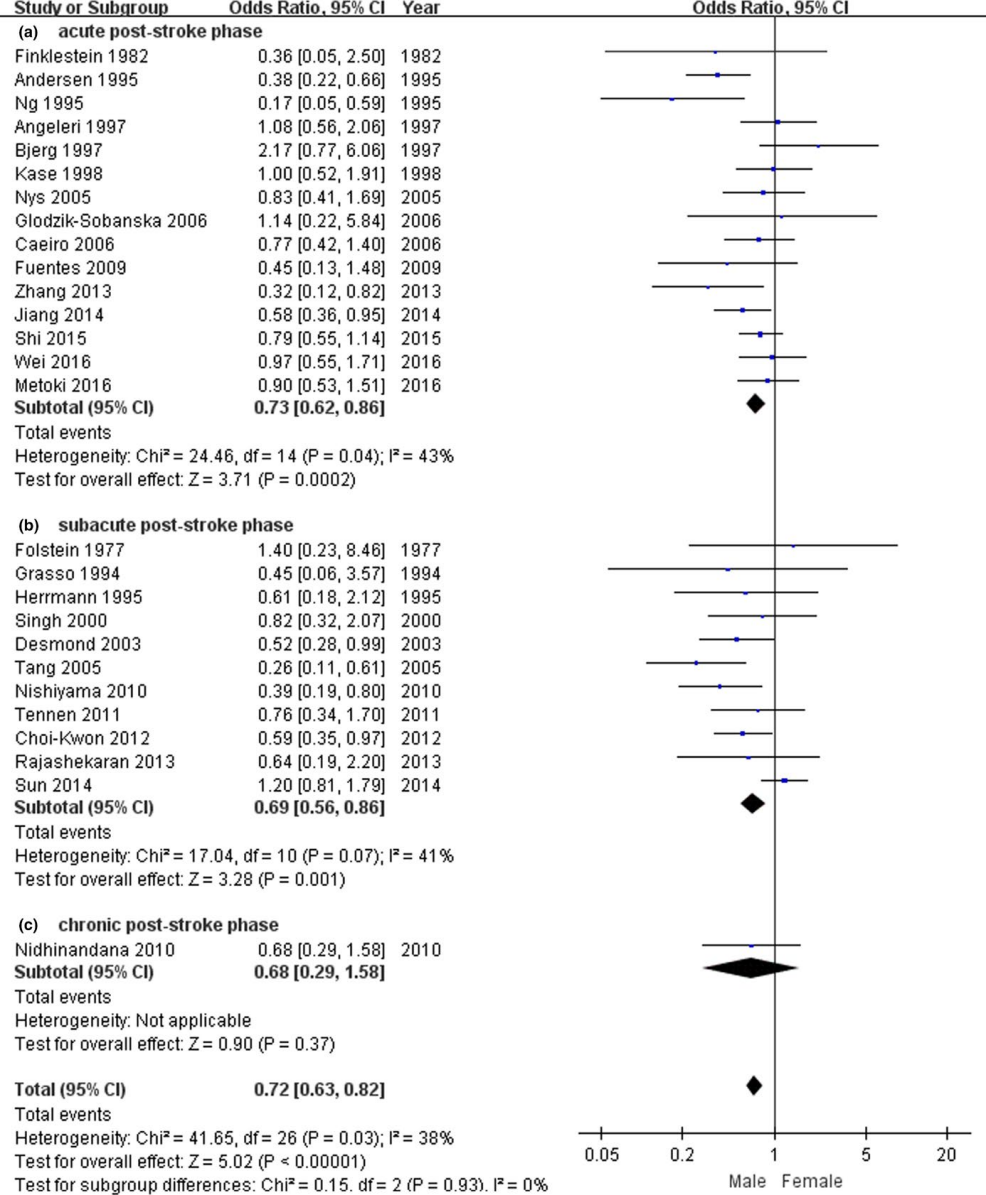
The forest plots of odds ratio with 95% confidence interval for the association between gender and depression risk according to time since stroke onset (*n* = 27)

In assessing the relationship between the sex and the prevalence of PSD, our results showed that there were significant heterogeneity in acute poststroke group (*n* = 15, *p*_heterogeneity_ = .04, *I* ^2^ = 43%, Figure 4a), and subacute poststroke group (*n* = 11, *p*_heterogeneity_ = .07, *I* ^2^ = 41%, Figure 4b). Thus, the random‐effects model was performed to synthesize the data. There was a statistically significant association between PSD and female stroke for the acute poststroke group (OR = 0.73, 95% CI 0.62–0.86, Figure 4b) and the subacute poststroke group (OR = 0.69, 95% CI 0.56–0.86, Figure 4b). Our results indicated that female stroke patients may be more susceptible to PSD during acute and subacute phase of stroke. Because the study of chronic poststroke group in this meta‐analysis only included 1 study (Figure 4c), it did not reveal any associations.

### 3.5 Quality assessment and sensitivity analyses

Quality assessment was provided in Table 1. The sensitive analyses were performed to assess the influence of any single study on the pooled OR. As shown in Table S1 and Table S2, our results showed that the corresponding ORs were not materially changed when any single study was omitted.

### 3.6 Publication bias

**Figure 5:**
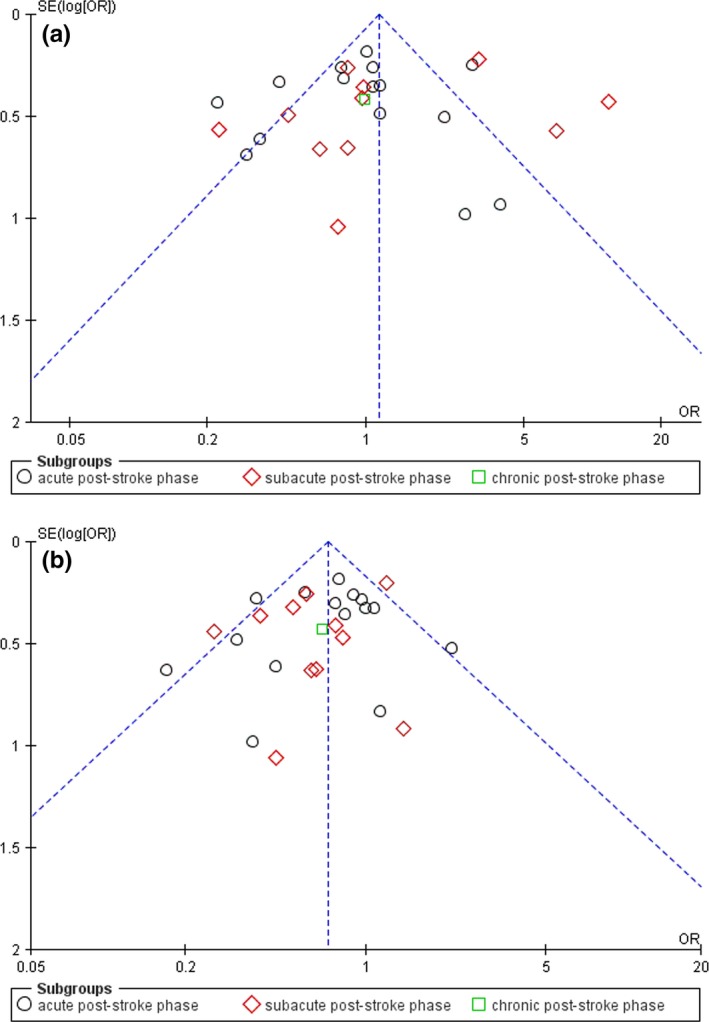
Funnel plot was designed to visualize a potential publication bias according to time since stroke onset (*n* = 27) (a) representing a potential publication bias for developing post‐stroke depression (PSD) after left hemisphere stroke. (b) representing a potential publication bias for developing PSD after male stroke

As shown in Figure 5, our results showed that no obvious asymmetry was found in the assessment of the funnel plots. No statistically significant differences were shown in the Egger's test analyses (*p* > .05).

## 4 DISCUSSION

PSD has proved to be a common sequela of stroke. Available clinical data about whether the risk of PSD is influenced by the location of the brain lesion and sex are unclear. Our meta‐analysis revealed that a significant association was found between the presence of PSD and left hemisphere lesions in subacute poststroke patients and PSD appears to be more susceptible to women PSD than men during acute and subacute poststroke phases.

### 4.1 Limitations of meta‐analysis in this study

There are several potential limitations in this study. Firstly, there was the possibility of information and selection biases and unidentified confounders because all available clinical data were from studies with the full text published in English. Secondly, although the brain stroke lesion and sex were significantly correlated with PSD, it is difficult to determine whether PSD is due to the clinical consequences of stroke. In future studies, we should consider to compare the control group, stroke group with no depression, and PSD to investigate the issue. Finally, it varied widely for the scales for depression assessment across different studies, and it was also not unified in the cut‐off points for different degrees of depressive symptoms.

### 4.2 Development of an appropriate and standardized measure of depression

It is difficult to measure the depression symptom in stroke patients. The phenomenology of PSD should be considered to be different from the noncomorbid depression. The measurement of PSD would take into account the impairments of function, cognition, and language that often accompany stroke (Bhogal et al., 2004; Wei et al., 2015). For example, stroke was considered to directly lead to the clinical symptoms of psychomotor retardation, fatigue, and sleep and appetite disturbances. These symptoms were classified as depressive symptoms by most conventional scales, which may result in an increased false‐positive depression scores (Wei et al., 2015). Therefore, it needs to develop an appropriate and standardized measure of depression to diagnose PSD.

### 4.3 Optimal time after stroke onset to assessment for PSD

Growing evidence has indicated that different clinical PSD subtypes are classified according to the different time interval between the depression diagnosis and the stroke onset and may exist different pathological mechanisms (Wei et al., 2015). A large number of studies have reported that the time interval can influence the relationship between the occurrence of PSD and the location of brain stroke lesion. The ≤3 months acute poststroke significantly favored PSD occurring after left brain anterior lesions (Santos et al., 2009; Shimoda & Robinson, 1999). During the 3–6 months poststroke period, the PSD occurring could be influenced by the proximity of the lesion to the frontal pole in both hemispheres (Santos et al., 2009; Shimoda & Robinson, 1999). The 1–2 years chronic stroke patients significantly favored PSD occurring after left hemisphere lesion. Furthermore, the occurrence of PSD with right brain hemisphere lesion is related to the stroke lesion size and closely to the occipital pole (Santos et al., 2009; Shimoda & Robinson, 1999). Carson et al. (2000) have indicated that PSD is not associated with stroke lesion location. Narushima, Kosier, & Robinson (2003) have reported that the occurrence of PSD is associated with left frontal and left basal ganglia lesions when depression was assessed within 2 months after stroke. Yu et al. (2004) have reported that the occurrence of PSD is associated with right hemisphere lesions during 4–9 months after stroke. Two meta‐analysis studies from either acute or chronic stroke patients have reported no significant association between PSD and lesion location (Ayerbe, Ayis, Wolfe, & Rudd, 2013; Kutlubaev & Hackett, 2014). However, a recent meta‐analysis showed that PSD has been associated right hemisphere lesion during 1–6 months after stroke (Wei et al., 2015). Our conclusion revealed that the occurrence of PSD is associated with left hemisphere lesions in subacute poststroke patients. This supported our view that these variances in the association between PSD and lesion location can be explained by the stratification of gender. Therefore, it is plausible that these variances in the association between PSD and stroke lesion location are reflected in gender effect on the association.

In addition, Sinyor et al. (1986) have indicated that the sex differences in the frequency of PSD may be masked by the excluding patients, who had a history of psychiatric disorder. More especially, it is reported that there is a sex‐specific prevalence of PSD. And the sex discrepancy in the prevalence of PSD are considered to reflect these differences in rehabilitation and medical care (Poynter et al., 2009). In our systematic review, the occurrence of PSD appears to be more common among women than men in acute and subacute poststroke patients. Reasons for higher prevalence of women PSD may be explained that the general population have the greater prevalence, such as psychosocial inequities and genetic factors and may also be explained by recovery, differential support, and access to rehabilitation (Grace et al., 2005). Furthermore, women are considered to be more likely than men to be diagnosed with some psychiatric disorders (Bekhbat & Neigh, 2017). For instance, Paradiso & Robert (1998) suggest that the sex discrepancy may owe to the differences of brain functioning and organization and further suggest that the women PSD is related to left‐sided brain lesions, but not men PSD. In the women PSD, the younger age, a history of psychiatric disorder, and impaired cognition are considered to be the psychosocial risk factors (Lavie, Milani, Cassidy, & Gilliland, 1999). Future study is needed to determine which factors may explain the greater rate of the women PSD and assess whether sex‐specific differences should be considered to be include as a factor in response to treatment.

In conclusion, converging evidence suggests that in the future, a prospective cohort study will be best suited for assessing the association between lesion location and sex and PSD. The clinical implications of this systematic review include the need for screening and identification of PSD among female stroke patients according to the different time interval between the depression diagnosis and stroke onset, in particular, and then provision of appropriate treatment.

## CONFLICT OF INTEREST

The authors declare no competing financial interests.

## References

[brb3788-bib-0001] Andersen, G. , Vestergaard, K. , Ingemann‐Nielsen, M. , & Lauritzen, L. (1995). Risk factors for post‐stroke depression. Acta Psychiatrica Scand., 92, 193–198. 10.1111/j.1600-0447.1995.tb09567.x 7484197

[brb3788-bib-0002] Angeleri, F. , Angeleri, V. A. , Foschi, N. , Giaquinto, S. , Nolfe, G. , Saginario, A. , & Signorino, M. (1997). Depression after stroke: An investigation through catamnesis. Journal of Clinical Psychiatry, 58, 261–265. 9228892 10.4088/jcp.v58n0605

[brb3788-bib-0003] Ayerbe, L. , Ayis, S. , Rudd, A. G. , Heuschmann, P. U. , & Wolfe, C. D. (2011). Natural history, predictors, and associations of depression 5 years after stroke: The South London Stroke Register. Stroke, 42, 1907–1911. 21566241 10.1161/STROKEAHA.110.605808

[brb3788-bib-0004] Ayerbe, L. , Ayis, S. , Wolfe, C. D. , & Rudd, A. G. (2013). Natural history, predictors and outcomes of depression after stroke: Systematic review and meta‐analysis. British Journal of Psychiatry, 202, 14–21. 23284148 10.1192/bjp.bp.111.107664

[brb3788-bib-0005] Begg, C. B. , & Mazumdar, M. (1994). Operating characteristics of a rank correlation test for publication bias. Biometrics, 50, 1088–1101. 7786990

[brb3788-bib-0006] Bekhbat, M. , & Neigh, G. N. (2017). Sex differences in the neuro‐immune consequences of stress: Focus on depression and anxiety. Brain, Behavior, and Immunity, https://doi.org/10.1016/j.bbi.2017.02.006. [Epub ahead of print]. 10.1016/j.bbi.2017.02.006 PMC5559342 28216088

[brb3788-bib-0007] Bhogal, S. K. , Teasell, R. , Foley, N. , & Speechley, M. (2004). Lesion location and poststroke depression: Systematic review of the methodological limitations in the literature. Stroke, 35, 794–802. 14963278 10.1161/01.STR.0000117237.98749.26

[brb3788-bib-0008] Bjerg Bendsen, B. , Bjerg Bendsen, E. , Lauritzen, L. , Vilmar, T. , & Bech, P. (1997). Post‐stroke patients in rehabilitation. The relationship between biological impairment (CT scanning), physical disability and clinical depression. European Psychiatry: The Journal of the Association of European Psychiatrists, 12, 399–404. 19698561 10.1016/S0924-9338(97)83565-1

[brb3788-bib-0009] Brodaty, H. , Withall, A. , Altendorf, A. , & Sachdev, P. S. (2007). Rates of depression at 3 and 15 months poststroke and their relationship with cognitive decline: The Sydney Stroke Study. American Journal of Geriatric Psychiatry, 15, 477–486. 17545448 10.1097/JGP.0b013e3180590bca

[brb3788-bib-0010] Caeiro, L. , Ferro, J. M. , Santos, C. O. , & Figueira, M. L. (2006). Depression in acute stroke. Journal of Psychiatry and Neuroscience, 31, 377–383. 17136215 PMC1635801

[brb3788-bib-0011] Carson, A. J. , MacHale, S. , Allen, K. , Lawrie, S. M. , Dennis, M. , House, A. , & Sharpe, M. (2000). Depression after stroke and lesion location: A systematic review. Lancet, 356, 122–126. 10963248 10.1016/S0140-6736(00)02448-X

[brb3788-bib-0012] Choi‐Kwon, S. , Han, K. , Choi, S. , Suh, M. , Kim, Y. J. , Song, H ., … Kim, J. S . (2012). Poststroke depression and emotional incontinence: Factors related to acute and subacute stages. Neurology, 78, 1130–1137. 22459674 10.1212/WNL.0b013e31824f8090 PMC3320054

[brb3788-bib-0013] Cully, J. A. , Gfeller, J. D. , Heise, R. A. , Ross, M. J. , Teal, C. R. , & Kunik, M. E. (2005). Geriatric depression, medical diagnosis, and functional recovery during acute rehabilitation. Archives of Physical Medicine and Rehabilitation, 86, 2256–2260. 16344020 10.1016/j.apmr.2005.07.292

[brb3788-bib-0014] Desmond, D. W. , Remien, R. H. , Moroney, J. T. , Stern, Y. , Sano, M. , & Williams, J. B. (2003). Ischemic stroke and depression. Journal of the International Neuropsychological Society, 9, 429–439. 12666767 10.1017/S1355617703930086

[brb3788-bib-0015] Egger, M. , Davey Smith, G. , Schneider, M. , & Minder, C. (1997). Bias in meta‐analysis detected by a simple, graphical test. BMJ, 315, 629–634. 9310563 10.1136/bmj.315.7109.629 PMC2127453

[brb3788-bib-0016] Esparrago Llorca, G. , Castilla‐Guerra, L. , Fernandez Moreno, M. C. , Ruiz Doblado, S. , & Jimenez Hernandez, M. D. (2015). Post‐stroke depression: An update. Neurologia, 30, 23–31. 22901370 10.1016/j.nrl.2012.06.008

[brb3788-bib-0017] Feibel, J. H. , & Springer, C. J. (1982). Depression and failure to resume social activities after stroke. Archives of Physical Medicine and Rehabilitation, 63, 276–277. 7082155

[brb3788-bib-0018] Finklestein, S. , Benowitz, L. I. , Baldessarini, R. J. , Arana, G. W. , Levine, D. , Woo, E. , … Stoll, A. L . (1982). Mood, vegetative disturbance, and dexamethasone suppression test after stroke. Annals of Neurology, 12, 463–468. 6960804 10.1002/ana.410120509

[brb3788-bib-0019] Folstein, M. F. , Maiberger, R. , & McHugh, P. R. (1977). Mood disorder as a specific complication of stroke. Journal of Neurology, Neurosurgery and Psychiatry, 40, 1018–1020. 10.1136/jnnp.40.10.1018 PMC492887 591971

[brb3788-bib-0020] Fuentes, B. , Ortiz, X. , Sanjose, B. , Frank, A. , & Diez‐Tejedor, E. (2009). Post‐stroke depression: Can we predict its development from the acute stroke phase? Acta Neurologica Scandinavica, 120, 150–156. 19154533 10.1111/j.1600-0404.2008.01139.x

[brb3788-bib-0021] Gainotti, G. , Antonucci, G. , Marra, C. , & Paolucci, S. (2001). Relation between depression after stroke, antidepressant therapy, and functional recovery. Journal of Neurology, Neurosurgery and Psychiatry, 71, 258–261. 10.1136/jnnp.71.2.258 PMC1737529 11459907

[brb3788-bib-0022] Gillen, R. , Tennen, H. , McKee, T. E. , Gernert‐Dott, P. , & Affleck, G. (2001). Depressive symptoms and history of depression predict rehabilitation efficiency in stroke patients. Archives of Physical Medicine and Rehabilitation, 82, 1645–1649. 11733876 10.1053/apmr.2001.26249

[brb3788-bib-0023] Glodzik‐Sobanska, L. , Slowik, A. , McHugh, P. , Sobiecka, B. , Kozub, J. , Rich, K. E. , … Szczudlik, A . (2006). Single voxel proton magnetic resonance spectroscopy in post‐stroke depression. Psychiatry Research, 148, 111–120. 17088051 10.1016/j.pscychresns.2006.08.004

[brb3788-bib-0024] Grace, S. L. , Abbey, S. E. , Pinto, R. , Shnek, Z. M. , Irvine, J. , & Stewart, D. E. (2005). Longitudinal course of depressive symptomatology after a cardiac event: Effects of gender and cardiac rehabilitation. Psychosomatic Medicine, 67, 52–58. 15673624 10.1097/01.psy.0000151486.28349.70 PMC2928242

[brb3788-bib-0025] Grasso, M. G. , Pantano, P. , Ricci, M. , Intiso, D. F. , Pace, A. , Padovani, A ., … Lenzi, G. L . (1994). Mesial temporal cortex hypoperfusion is associated with depression in subcortical stroke. Stroke, 5, 980–985. 10.1161/01.str.25.5.980 8165694

[brb3788-bib-0026] Gu, L. , Su, L. , Wu, G. , Chen, Q. , Yan, Y. , Xie, J. , … Tang, N . (2013). Association between TNF‐delta 238G/A polymorphisms and the risk of ischemic stroke. International Journal of Neuroscience, 123, 1–6. 22937788 10.3109/00207454.2012.725118

[brb3788-bib-0027] Hadidi, N. , Treat‐Jacobson, D. J. , & Lindquist, R. (2009). Poststroke depression and functional outcome: A critical review of literature. Heart and Lung, 38, 151–162. 19254633 10.1016/j.hrtlng.2008.05.002

[brb3788-bib-0028] Herrmann, M. , Bartels, C. , Schumacher, M. , & Wallesch, C. W. (1995). Poststroke depression. Is there a pathoanatomic correlate for depression in the postacute stage of stroke? Stroke, 26, 850–856. 7740579 10.1161/01.str.26.5.850

[brb3788-bib-0029] Hsieh, L. P. , & Kao, H. J. (2005). Depressive symptoms following ischemic stroke: A study of 207 patients. Acta Neurologica Taiwanica, 14, 187–190. 16425545

[brb3788-bib-0030] Jiang, X. G. , Lin, Y. , & Li, Y. S. (2014). Correlative study on risk factors of depression among acute stroke patients. European Review for Medical and Pharmacological Sciences, 18, 1315–1323. 24867509

[brb3788-bib-0031] Kase, C. S. , Wolf, P. A. , Kelly‐Hayes, M. , Kannel, W. B. , Beiser, A. , & D'Agostino, R. B. (1998). Intellectual decline after stroke: The Framingham Study. Stroke, 29, 805–812. 9550515 10.1161/01.str.29.4.805

[brb3788-bib-0032] Kutlubaev, M. A. , & Hackett, M. L. (2014). Part II: Predictors of depression after stroke and impact of depression on stroke outcome: An updated systematic review of observational studies. International Journal of Stroke, 9, 1026–1036. 25156411 10.1111/ijs.12356

[brb3788-bib-0033] Lavie, C. J. , Milani, R. V. , Cassidy, M. M. , & Gilliland, Y. E. (1999). Effects of cardiac rehabilitation and exercise training programs in women with depression. American Journal of Cardiology, 3, 1480–1483, A7. 10.1016/s0002-9149(99)00127-7 10335766

[brb3788-bib-0034] MacHale, S. M. , O'Rourke, S. J. , Wardlaw, J. M. , & Dennis, M. S. (1998). Depression and its relation to lesion location after stroke. Journal of Neurology, Neurosurgery and Psychiatry, 64, 371–374. 10.1136/jnnp.64.3.371 PMC2170020 9527152

[brb3788-bib-0035] Mackay, J. , & Mensah, G. (2004). The atlas of heart disease and stroke. Geneva, Switzerland: World Health Organization.

[brb3788-bib-0036] Mayberg, H. S. , Robinson, R. G. , Wong, D. F. , Parikh, R. , Bolduc, P. , Starkstein, S. E. , … Wilson, A. A. (1988). PET imaging of cortical S2 serotonin receptors after stroke: Lateralized changes and relationship to depression. American Journal of Psychiatry, 145, 937–943. 3394877 10.1176/ajp.145.8.937

[brb3788-bib-0037] Metoki, N. , Sugawara, N. , Hagii, J. , Saito, S. , Shiroto, H. , Tomita, T ., … Yasui‐Furukori, N . (2016). Relationship between the lesion location of acute ischemic stroke and early depressive symptoms in Japanese patients. Annals of General Psychiatry, 15, 12. 27042194 10.1186/s12991-016-0099-x PMC4818403

[brb3788-bib-0038] Narushima, K. , Kosier, J. T. , & Robinson, R. G. (2003). A reappraisal of poststroke depression, intra‐ and inter‐hemispheric lesion location using meta‐analysis. Journal of Neuropsychiatry and Clinical Neurosciences, 15, 422–430. 14627768 10.1176/jnp.15.4.422

[brb3788-bib-0039] Ng, K. C. , Chan, K. L. , & Straughan, P. T. (1995). A study of post‐stroke depression in a rehabilitative center. Acta Psychiatrica Scand., 92, 75–79. 10.1111/j.1600-0447.1995.tb09546.x 7572253

[brb3788-bib-0040] Nidhinandana, S. , Sithinamsuwan, P. , Chinvarun, Y. , Wongmek, W. , Supakasem, S. , & Suwantamee, J. (2010). Prevalence of poststroke depression in Thai stroke survivors studied in Phramongkutklao Hospital. Journal of the Medical Association of Thailand, 93(Suppl 6), S60–S64. 21280517

[brb3788-bib-0041] Nishiyama, Y. , Komaba, Y. , Ueda, M. , Nagayama, H. , Amemiya, S. , & Katayama, Y. (2010). Early depressive symptoms after ischemic stroke are associated with a left lenticulocapsular area lesion. Journal of Stroke and Cerebrovascular Diseases: the Official Journal of National Stroke Association, 19, 184–189. 20434044 10.1016/j.jstrokecerebrovasdis.2009.04.002

[brb3788-bib-0042] Nys, G. M. , van Zandvoort, M. J. , van der Worp, H. B. , de Haan, E. H. , de Kort, P. L. , & Kappelle, L. J. (2005). Early depressive symptoms after stroke: Neuropsychological correlates and lesion characteristics. Journal of the Neurological Sciences, 228, 27–33. 15607207 10.1016/j.jns.2004.09.031

[brb3788-bib-0043] Paradiso, S. , & Robert, R. (1998). Gender differences in poststroke depression. Journal of Neuropsychiatry and Clinical Neurosciences, 10, 41–47. 9547465 10.1176/jnp.10.1.41

[brb3788-bib-0044] Parikh, R. M. , Robinson, R. G. , Lipsey, J. R. , Starkstein, S. E. , Fedoroff, J. P. , & Price, T. R. (1990). The impact of poststroke depression on recovery in activities of daily living over a 2‐year follow‐up. Archives of Neurology, 47, 785–789. 2357159 10.1001/archneur.1990.00530070083014

[brb3788-bib-0045] Poynter, B. , Shuman, M. , Diaz‐Granados, N. , Kapral, M. , Grace, S. L. , & Stewart, D. E. (2009). Sex differences in the prevalence of post‐stroke depression: A systematic review. Psychosomatics, 50, 563–569. 19996226 10.1176/appi.psy.50.6.563

[brb3788-bib-0046] Rajashekaran, P. , Pai, K. , Thunga, R. , & Unnikrishnan, B. (2013). Post‐stroke depression and lesion location: A hospital based cross‐sectional study. Indian Journal of Psychiatry, 55, 343–348. 24459304 10.4103/0019-5545.120546 PMC3890916

[brb3788-bib-0047] RevMan (2011). Review manager (RevMan), version 5.1. Copenhagen: The Nordic Cochrane Centre, The Cochrane Collaboration.

[brb3788-bib-0048] Robinson, R. G. , & Jorge, R. E. (2016). Post‐stroke depression: A review. American Journal of Psychiatry, 173, 221–231. 26684921 10.1176/appi.ajp.2015.15030363

[brb3788-bib-0049] Robinson, R. G. , Kubos, K. L. , Starr, L. B. , Rao, K. , & Price, T. R. (1983). Mood changes in stroke patients: Relationship to lesion location. Comprehensive Psychiatry, 24, 555–566. 6653097 10.1016/0010-440x(83)90024-x

[brb3788-bib-0050] Robinson, R. G. , Shoemaker, W. J. , Schlumpf, M. , Valk, T. , & Bloom, F. E. (1975). Effect of experimental cerebral infarction in rat brain on catecholamines and behaviour. Nature, 255, 332–334. 1128692 10.1038/255332a0

[brb3788-bib-0051] Robinson, R. G. , Starr, L. B. , Kubos, K. L. , & Price, T. R. (1983). A two‐year longitudinal study of post‐stroke mood disorders: Findings during the initial evaluation. Stroke, 14, 736–741. 6658957 10.1161/01.str.14.5.736

[brb3788-bib-0052] Santos, M. , Kovari, E. , Gold, G. , Bozikas, V. P. , Hof, P. R. , Bouras, C. , & Giannakopoulos, P. (2009). The neuroanatomical model of post‐stroke depression: Towards a change of focus? Journal of the Neurological Sciences, 283, 158–162. 19264329 10.1016/j.jns.2009.02.334 PMC2915758

[brb3788-bib-0053] Saxena, A. , & Suman, A. (2015). Magnitude and determinants of depression in acute stroke patients admitted in a rural tertiary care hospital. Journal of Neurosciences in Rural Practice, 6, 202–207. 25883481 10.4103/0976-3147.153228 PMC4387812

[brb3788-bib-0054] Shi, Y. , Xiang, Y. , Yang, Y. , Zhang, N. , Wang, S. , Ungvari, G. S ., … Wang, C . (2015). Depression after minor stroke: Prevalence and predictors. Journal of Psychosomatic Research, 79, 143–147. 25868687 10.1016/j.jpsychores.2015.03.012

[brb3788-bib-0055] Shimoda, K. , & Robinson, R. G. (1999). The relationship between poststroke depression and lesion location in long‐term follow‐up. Biological Psychiatry, 45, 187–192. 9951566 10.1016/s0006-3223(98)00178-4

[brb3788-bib-0056] Singh, A. , Black, S. E. , Herrmann, N. , Leibovitch, F. S. , Ebert, P. L. , Lawrence, J. , & Szalai, J. P. (2000). Functional and neuroanatomic correlations in poststroke depression: The Sunnybrook Stroke Study. Stroke, 31, 637–644. 10700497 10.1161/01.str.31.3.637

[brb3788-bib-0057] Sinyor, D. , Jacques, P. , Kaloupek, D. G. , Becker, R. , Goldenberg, M. , & Coopersmith, H. (1986). Poststroke depression and lesion location. An attempted replication. Brain, 109(Pt 3), 537–546. 3719289 10.1093/brain/109.3.537

[brb3788-bib-0058] Sun, N. , Li, Q. J. , Lv, D. M. , Man, J. , Liu, X. S. , & Sun, M. L. (2014). A survey on 465 patients with post‐stroke depression in China. Archives of Psychiatric Nursing, 28, 368–371. 25457685 10.1016/j.apnu.2014.08.007

[brb3788-bib-0059] Tang, W. K. , Chan, S. S. , Chiu, H. F. , Ungvari, G. S. , Wong, K. S. , Kwok, T. C. , … Ahuja, A. T . (2005). Poststroke depression in Chinese patients: Frequency, psychosocial, clinical, and radiological determinants. Journal of Geriatric Psychiatry and Neurology, 18, 45–51. 15681628 10.1177/0891988704271764

[brb3788-bib-0060] Tennen, G. , Herrmann, N. , Black, S. E. , Levy, K. S. , Cappell, J. , Li, A. , & Lanctôt, K. L. (2011). Are vascular risk factors associated with post‐stroke depressive symptoms? Journal of Geriatric Psychiatry and Neurology, 24, 215–221. 22228828 10.1177/0891988711422526

[brb3788-bib-0061] Tsai, C. F. , Anderson, N. , Thomas, B. , & Sudlow, C. L. (2015). Risk factors for ischemic stroke and its subtypes in Chinese vs. Caucasians: Systematic review and meta‐analysis. International Journal of Stroke, 10, 485–493. 25907735 10.1111/ijs.12508

[brb3788-bib-0062] Tsai, C. F. , Anderson, N. , Thomas, B. , & Sudlow, C. L. (2016). Comparing risk factor profiles between intracerebral hemorrhage and ischemic stroke in Chinese and White populations: Systematic review and meta‐analysis. PLoS ONE, 11, e0151743. 26991497 10.1371/journal.pone.0151743 PMC4798495

[brb3788-bib-0063] Wei, N. , Yong, W. , Li, X. , Zhou, Y. , Deng, M. , Zhu, H. , & Jin, H. (2015). Post‐stroke depression and lesion location: A systematic review. Journal of Neurology, 262, 81–90. 25308633 10.1007/s00415-014-7534-1

[brb3788-bib-0064] Wei, C. , Zhang, F. , Chen, L. , Ma, X. , Zhang, N. , & Hao, J. (2016). Factors associated with post‐stroke depression and fatigue: Lesion location and coping styles. Journal of Neurology, 263, 269–276. 26568559 10.1007/s00415-015-7958-2 PMC4751197

[brb3788-bib-0065] Williams, L. S. , Ghose, S. S. , & Swindle, R. W. (2004). Depression and other mental health diagnoses increase mortality risk after ischemic stroke. American Journal of Psychiatry, 161, 1090–1095. 15169698 10.1176/appi.ajp.161.6.1090

[brb3788-bib-0066] Yu, L. , Liu, C. K. , Chen, J. W. , Wang, S. Y. , Wu, Y. H. , & Yu, S. H. (2004). Relationship between post‐stroke depression and lesion location: A meta‐analysis. Kaohsiung Journal of Medical Sciences, 20, 372–380. 15473648 10.1016/S1607-551X(09)70173-1 PMC11918149

[brb3788-bib-0067] Zhang, W. N. , Pan, Y. H. , Wang, X. Y. , & Zhao, Y. (2013). A prospective study of the incidence and correlated factors of post‐stroke depression in China. PLoS ONE, 8, e78981. 24260141 10.1371/journal.pone.0078981 PMC3832506

