# Supplementary material for: The association between lesion location, sex and poststroke depression: Meta‐analysis
Source: Brain Behav. 2017 Aug 30;7(10):e00788. doi: 10.1002/brb3.788 (PMC5651383; doi:10.1002/brb3.788)
Supplement: Supplementary file 1 [file BRB3-7-e00788-s001.docx]

# The association between lesion location, sex and post-stroke depression: meta-analysis

# Results

## Source Populations

Subgroups analyses were performed according to population source. In assessing the associations between stroke location and the prevalence of PSD, our results showed that there were two trends between clinic population source and rehabilitation population when studies were investigated according to population source (**Fig. S1**). Studies of clinic population source significantly favored PSD occurring after a left hemisphere stroke (OR = 1.26; 95% CI 1.10 – 1.45), whereas studies of rehabilitation population source showed a trend to favor PSD after a right hemisphere stroke (OR = 0.90; 95% CI 0.65 – 1.30). Because the study of community-based population only included 1 study, it did not reveal the association.

In assessing the associations between gender of stroke and the prevalence of PSD, our results showed that there were similar trends between clinic population source and rehabilitation population when studies were investigated according to population source (**Fig. S2**). Studies of clinic population source and rehabilitation population source significantly favored PSD occurring after female stroke (Clinic population source: OR=0.68; 95% CI 0.59 – 0.78; rehabilitation population source: OR = 0.71; 95% CI 0.62 – 0.80). Because the study of community-based population only included 1 study, it did not reveal the association.

Publication bias

As shown in **Figure S3**, the assessment of the funnel plots did not reveal any evidence of obvious asymmetry. The Egger’s test analyses also showed that there were not statistically significant differences (*P* > 0.05).

# Tables

## Table S1.

**Sensitivity analyses for overall studies of developing PSD after left hemisphere stroke.**

| Study omitted | OR (95% CI) | *I^2^*(%) | Study omitted | OR (95% CI) | *I^2^*(%) |
| --- | --- | --- | --- | --- | --- |
| Folstein 1977 | 1.10 (0.82, 1.49) | 78 | Glodzik-Sobanska 2006 | 1.08 (0.80,1.46) | 78 |
| Finklestein 1982 | 1.09 (0.81,1.47) | 78 | Brodaty 2007 | 1.12 (0.82,1.52) | 78 |
| Grasso 1994 | 1.11 (0.83,1.50) | 78 | Fuentes 2009 | 1.14 (0.85,1.54) | 78 |
| Herrmann 1995 | 1.12 (0.83,1.51) | 78 | Nidhinandana 2010 | 1.11 (0.82,1.51) | 78 |
| Andersen 1995 | 1.17 (0.88,1.57) | 76 | Nishiyama 2010 | 1.02 (0.78,1.34) | 72 |
| Ng 1995 | 1.14 (0.85,1.54) | 78 | Tennen 2011 | 1.11 (0.82,1.51) | 78 |
| Angeleri 1997 | 1.12 (0.83,1.53) | 78 | Choi-Kwon 2012 | 1.12 (0.82,1.53) | 78 |
| Bjerg 1997 | 1.08 (0.80,1.47) | 78 | Zhang 2013 | 1.11 (0.82,1.50) | 78 |
| Kase 1998 | 1.15 (0.86,1.55) | 77 | Rajashekaran 2013 | 1.05 (0.79,1.41) | 77 |
| Singh 2000 | 1.16 (0.87,1.56) | 77 | Sun 2014 | 1.06 (0.79,1.41) | 74 |
| Gainotti 2001 | 1.12 (0.83,1.52) | 78 | Jiang 2014 | 1.06 (0.79,1.43) | 76 |
| Desmond 2003 | 1.11 (0.82,1.51) | 78 | Shi 2015 | 1.11 (0.81,1.53) | 78 |
| Tang 2005 | 1.14 (0.85,1.31) | 78 | Saxena 2015 | 1.07 (0.79,1.44) | 77 |
| Hsieh 2005 | 1.11 (0.81,1.51) | 78 | Wei 2016 | 1.11 (0.81,1.51) | 78 |
| Nys 2005 | 1.11 (0.82,1.51) | 78 | Metoki 2016 | 1.12 (0.83,1.53) | 78 |
| Caeiro 2006 | 1.11 (0.81,1.50) | 78 |  |  |  |

For all of the studies, P_heterogeneity_ < 0.00001.

## Table S2.

**Sensitivity analyses for overall studies of developing PSD after male stroke.**

| Study omitted | OR (95% CI) | *I^2^*(%) | Study omitted | OR (95% CI) | *I^2^*(%) |
| --- | --- | --- | --- | --- | --- |
| Folstein 1977 | 0.68 (0.58, 0.80) | 34 | Glodzik-Sobanska 2006 | 0.68 (0.58,0.80) | 78 |
| Finklestein 1982 | 0.69 (0.58, 0.81) | 35 | Brodaty 2007 | 0.69 (0.58,0.81) | 78 |
| Grasso 1994 | 0.69 (0.58,0.81) | 35 | Fuentes 2009 | 0.69 (0.58,0.81) | 78 |
| Herrmann 1995 | 0.69 (0.58,0.81) | 35 | Nidhinandana 2010 | 0.68 (0.58,0.81) | 78 |
| Andersen 1995 | 0.71 (0.60,0.83) | 27 | Nishiyama 2010 | 0.70 (0.59,0.82) | 72 |
| Ng 1995 | 0.70 (0.60,0.82) | 27 | Tennen 2011 | 0.68 (0.58,0.81) | 78 |
| Angeleri 1997 | 0.67 (0.57,0.79) | 33 | Choi-Kwon 2012 | 0.69 (0.58,0.82) | 78 |
| Bjerg 1997 | 0.67 (0.57,0.79) | 28 | Zhang 2013 | 0.70 (0.58,0.81) | 78 |
| Kase 1998 | 0.67 (0.57,0.80) | 34 | Rajashekaran 2013 | 0.68 (0.79,1.41) | 77 |
| Singh 2000 | 0.68 (0.58,0.81) | 35 | Sun 2014 | 0.66 (0.56,0.77) | 74 |
| Gainotti 2001 | 0.68 (0.58,0.80) | 35 | Jiang 2014 | 0.69 (0.58,0.82) | 76 |
| Desmond 2003 | 0.69 (0.58,0.82) | 34 | Shi 2015 | 0.68 (0.57,0.80) | 78 |
| Tang 2005 | 0.71 (0.60,0.83) | 27 | Saxena 2015 | 0.68 (0.57,0.80) | 77 |
| Hsieh 2005 | 0.70 (0.59,0.82) | 32 | Wei 2016 | 0.67 (0.57,0.80) | 78 |
| Nys 2005 | 0.68 (0.57,0.80) | 35 | Metoki 2016 | 0.67 (0.57,0.80) | 78 |
| Caeiro 2006 | 0.68 (0.57,0.81) | 35 |  |  |  |

For all of the studies, P_heterogeneity_ < 0.00001.

Table S4. Sensitivity analyses for subgroups according to the time since stroke onset to assessment for PSD.

| Study omitted | OR (95% CI) | *I^2^*(%) | Study omitted | OR (95% CI) | *I^2^*(%) |
| --- | --- | --- | --- | --- | --- |
| **Acute** |  |  | **Subacute** |  |  |
| Robinson, R. G. | 1.16 (0.85,1.59) | 63.15 | Folstein, M. F. | 0.74 (0.58,0.95) | 34.66 |
| House, A. | 1.24 (0.90,1.71) | 67.61 | Eastwood, M. R. | 0.74 (0.58,0.95) | 35.97 |
| Starkstein, S. E. | 1.15 (0.84,1.57) | 60.82 | House, A. | 0.75 (0.59,0.96) | 33.09 |
| Astrom, M. | 1.10 (0.84,1.45) | 43.78 | Morris, P. L. | 0.72 (0.56,0.93) | 35.31 |
| Iacoboni, M. | 1.22 (0.88,1.68) | 68.03 | Astrom, M. | 0.77 (0.61,0.97) | 28.80 |
| Andersen, G. | 1.27 (0.92,1.76) | 65.48 | Herrmann, M. | 0.73 (0.57,0.94) | 35.42 |
| Gonzalez-Torrecillas | 1.17 (0.77,1.79) | 68.51 | MacHale, S. M | 0.78 (0.62,0.98) | 25.66 |
| Shimoda, K. | 1.25 (0.89,1.74) | 29.79 | Kase, C. S. | 0.76 (0.59,0.97) | 32.26 |
| Berg, A | 1.10 (0.82,1.46) | 64.14 | Pohjasvaara, T. | 0.71 (0.56, 0.90) | 20.38 |
| Nys, G. M. | 1.16 (0.85,1.60) | 59.46 | Paolucci, S | 0.75 (0.58,0.98) | 33.78 |
| Caeiro, L. | 1.29 (0.94,1.76) | 69.19 | Singh, A | 0.79 (0.63, 0.99) | 21.78 |
| Glodzik-Sobanska | 1.23 (0.90,1.70) | 67.73 | Gainotti, G. | 0.78 (0.63,0.97) | 34.20 |
| Provinciali, L. | 1.24 (0.86,1.79) | 64.46 | Desmond, D. W. | 0.73 (0.56,0.94) | 36.80 |
| Nishiyama, Y. | 1.22 (0.87,1.71) | 68.73 | Spalletta, G. | 0.72 (0.56, 0.93) | 36.24 |
| Bour, A. | 1.24 (0.89,1.73) | 68.73 | Tang, W. K. | 0.74 (0.58, 0.95) | 35.51 |
| Altieri, M. | 1.25 (0.90,1.75) | 68.16 | Glodzik-Sobanska | 0.73 (0.58,0.93) | 33.22 |
| Choi-Kwon, S. | 1.26 (0.90,1.78) | 66.72 | Oladiji, J. O. | 0.76 (0.59,0.97) | 32.11 |
| Zhang, W. N. | 1.23 (0.89,1.71) | 68.95 | Fuentes, B. | 0.76 (0.60, 0.97) | 30.05 |
|  |  |  | Snaphaan, L. | 0.71 (0.56,0.91) | 25.89 |
| **Chronic** |  |  | Oladiji, J. O. | 0.76 (0.59,0.89) | 32.11 |
| House, A. | 0.84 (0.47,1.52) | 0.00 | Tennen, G. | 0.73 (0.57,0.94) | 36.81 |
| Sharpe, M. | 0.70 (0.37,1.31) | 0.00 | Choi-Kwon, S. | 0.73 (0.56,0.95) | 37.59 |
| Astrom, M. | 0.71 (0.39,1.32) | 0.00 |  |  |  |
| Andersen, G. | 0.87 (0.49,1.57) | 0.00 |  |  |  |
| Iacoboni, M. | 0.76 (0.42,1.37) | 0.00 |  |  |  |
| Nidhinandana, S. | 0.69 (0.32,1.48) | 0.00 |  |  |  |

For all of the studies, *P*_heterogeneity_ < 0.1.

# Figures legends

## Figure S1


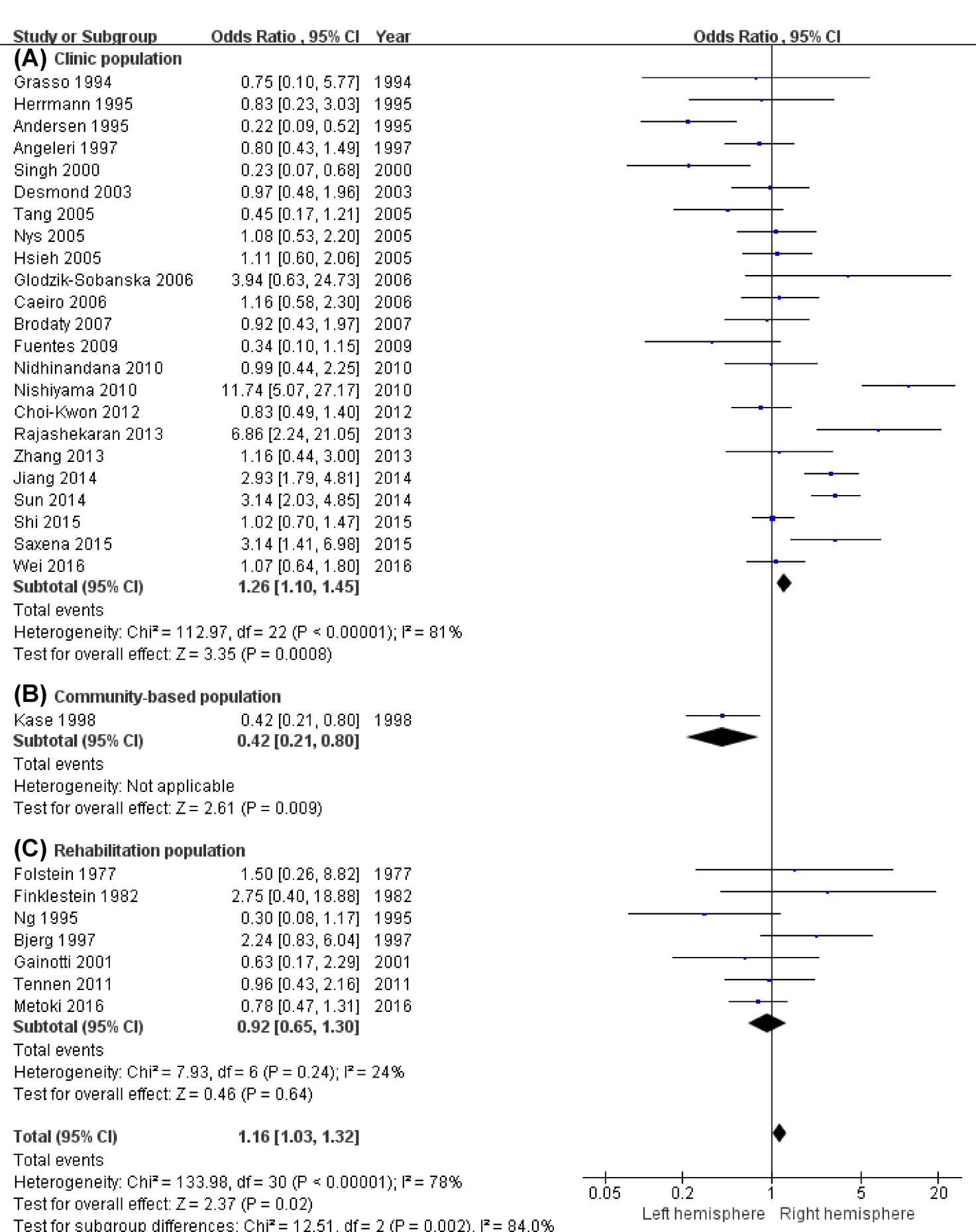


**Figure S1 The forest plots of OR with 95 % CI for the association between lesion location and depression risk according to patient source (n=31)**

## Figure S2


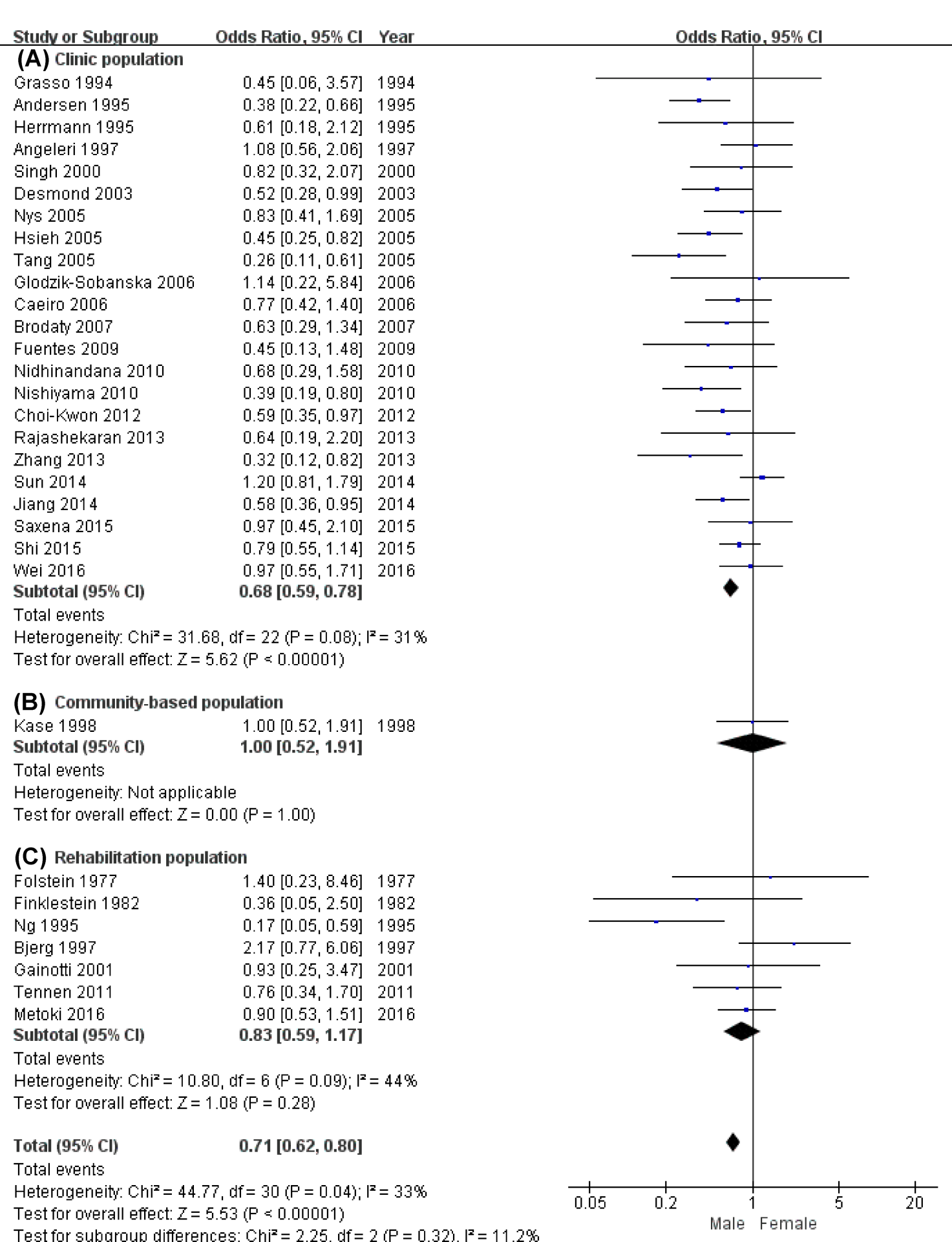


**Figure S2 The forest plots of OR with 95 % CI for the association between gender and depression risk according to patient source (n=31)**

## Figure S3


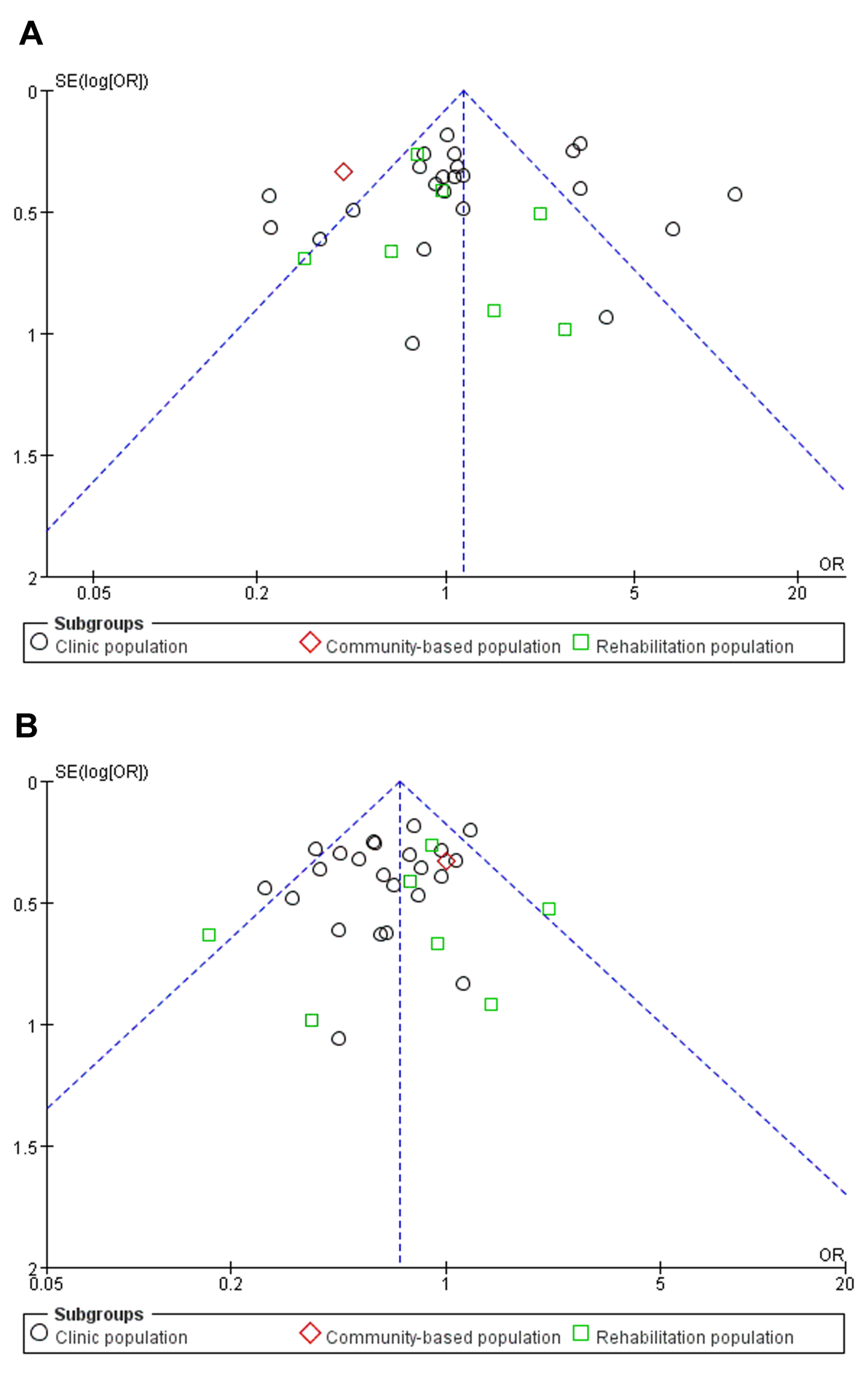


**Figure S3 Funnel plot was designed to visualize a potential publication bias according to patient source (n=31).**

1. **representing a potential publication bias for developing PSD after left hemisphere stroke .**
2. **representing a potential publication bias for developing PSD after male stroke.**
